# MAGI-1 Modulates AMPA Receptor Synaptic Localization and Behavioral Plasticity in Response to Prior Experience

**DOI:** 10.1371/journal.pone.0004613

**Published:** 2009-02-26

**Authors:** Lesley Emtage, Howard Chang, Rebecca Tiver, Christopher Rongo

**Affiliations:** The Waksman Institute, Department of Genetics, Rutgers University, Piscataway, New Jersey, United States of America; Pennsylvania State University, United States of America

## Abstract

It is well established that the efficacy of synaptic connections can be rapidly modified by neural activity, yet how the environment and prior experience modulate such synaptic and behavioral plasticity is only beginning to be understood. Here we show in *C. elegans* that the broadly conserved scaffolding molecule MAGI-1 is required for the plasticity observed in a glutamatergic circuit. This mechanosensory circuit mediates reversals in locomotion in response to touch stimulation, and the AMPA-type receptor (AMPAR) subunits GLR-1 and GLR-2, which are required for reversal behavior, are localized to ventral cord synapses in this circuit. We find that animals modulate GLR-1 and GLR-2 localization in response to prior mechanosensory stimulation; a specific isoform of MAGI-1 (MAGI-1L) is critical for this modulation. We show that MAGI-1L interacts with AMPARs through the intracellular domain of the GLR-2 subunit, which is required for the modulation of AMPAR synaptic localization by mechanical stimulation. In addition, mutations that prevent the ubiquitination of GLR-1 prevent the decrease in AMPAR localization observed in previously stimulated *magi-1* mutants. Finally, we find that previously-stimulated animals later habituate to subsequent mechanostimulation more rapidly compared to animals initially reared without mechanical stimulation; MAGI-1L, GLR-1, and GLR-2 are required for this change in habituation kinetics. Our findings demonstrate that prior experience can cause long-term alterations in both behavioral plasticity and AMPAR localization at synapses in an intact animal, and indicate a new, direct role for MAGI/S-SCAM proteins in modulating AMPAR localization and function in the wake of variable sensory experience.

## Introduction

Experience leads to learning and memory formation through changes in the strength of connectivity between neurons (synaptic plasticity). The regulated trafficking of AMPARs at synapses is an important mechanism for achieving synaptic plasticity [1], [2]. How actual experience modulates AMPAR trafficking, particularly *in vivo*, remains a topic of intense study.

The molecular mechanisms that mediate synaptic plasticity are beginning to be elucidated; many of the key components involved are synaptic scaffolding molecules of the membrane-associated guanylate kinase (MAGUK) family such as PSD-95 and PSD-93 [1], [2]. Closely related to the MAGUK family of scaffolding molecules are the inverted MAGUKs (MAGIs). Mammalian MAGIs are found at both synapses and epithelial junctions [3]–[5]. MAGI-2 (also called synaptic scaffolding molecule, or S-SCAM) has been shown to interact with NMDA receptors at excitatory synapses [4]. This member of the MAGI subfamily of MAGUKs has three splice variants: α, β, and γ [6]. In mice, deletions specific to the α isoform are lethal within 24 hours after birth, and affect dendritic spine morphology [7]. Indeed, mammalian MAGI-2 has been shown to interact with a great variety of post-synaptic density proteins; however, little is known about the precise function of MAGI-2 at excitatory synapses.

The function of specific synaptic scaffolding molecules in AMPAR trafficking and synaptic plasticity can be addressed in intact neural circuits using *C. elegans*, which use the AMPAR-like subunits GLR-1 and GLR-2 [8]–[10]. Both are expressed in the command interneurons that drive all forward and backward motion [11]. These neurons receive input from mechanosensory neurons, forming a reversal circuit that allows animals to escape from harm [12]. This reversal circuit also governs periodic, spontaneous reversals in locomotion, which are probably a part of the foraging strategy for covering maximum area [13]. GLR-1 and GLR-2 are required for these spontaneous reversals in locomotion [8], [9], [13], [14]

*C. elegans* can learn and remember prior mechanosensory stimulation and modulate their behavior accordingly [15]. The reversal circuit can be activated by non-directional taps to their culture dish; this tap response is mediated by a combination of gap junctions and chemical synapses between mechanosensory neurons and their command interneuron targets [12], [16], [17]. Animals habituate after repeated taps, showing a diminished magnitude of reversal distance with each tap [17]. If they experience multiple, spaced periods of stimulation, animals will also exhibit long-term habituation, exhibiting a diminished reversal magnitude in response to subsequent tap stimulation up to 24 hours later [17], [18]. GLR-1 is required for this long-term memory, and prior stimulation alters the size of GLR-1 clusters in the posterior ventral nerve cord, suggesting that prior experience alters GLR-1 expression or localization [19].

GLR-1 has been shown to form a complex with the AMPAR subunit GLR-2 [8], [9], [13], [14]. Interestingly, while GLR-1 is absolutely required for a rapid, glutamate-gated current in the command interneurons, the same neurons from *glr-2* null animals show some residual current. These findings imply that AMPARs are not functional in the absence of GLR-1, and that AMPARs in *glr-2* mutants contain only GLR-1 homomers. GLR-1/GLR-2 heteromers predominate in wild-type animals, with some additional contribution by GLR-1 homomers [10].

Fluorescently tagged versions of GLR-1 and GLR-2 are localized to synapses along the ventral nerve cord; this localization is regulated by ubiquitination and membrane recycling [10], [14], [20]–[25]. The cytoplasmic domain of GLR-2 is sufficient, when added to a transmembrane domain, to mediate such trafficking and synaptic localization [14]. However, both behavioral and electrophysiological data show that GLR-2 does not form homomeric channels. Taken together, these data suggest that sequences in the GLR-2 tail mediate the trafficking and localization of GLR-1/GLR-2 heteromeric channels. The proteins that interact with these GLR-2 tail sequences have not yet been identified.

Here we show that a specific isoform of the scaffolding molecule MAGI-1 (MAGI-1L), a close homolog of mammalian MAGI/S-SCAM proteins, interacts with AMPA receptors through binding to the GLR-2 subunit. We also show that prior mechanosensory stimulation alters AMPAR localization in the interneurons that connect mechanosensory input with motor output; such alterations require MAGI-1L function. Specifically, we used a GFP fusion to examine GLR-1 localization both before and after mechanical stimulation, and we find that wild-type *C. elegans* roughly maintain consistent synaptic GLR-1 localization regardless of prior mechanosensory stimulation, whereas stimulated mutants lacking MAGI-1L show a reduction in localized GLR-1. In addition, we find that wild-type animals show a mechanosensory stimulation-dependent change in GLR-2 localization; this change also requires MAGI-1L. The requirement for MAGI-1L function in regulating GLR-1 localization is abolished in mutants lacking GLR-2, suggesting that MAGI-1L responds to prior experience by specifically modulating the trafficking of GLR-1/GLR-2 heteromers via its interaction with GLR-2. Finally, *C. elegans* exhibit a form of long-term memory in response to repetitive mechanical stimulation that requires GLR-1 [19]; we find that both GLR-2 and MAGI-1L mutants share a similar defect in long-term memory acquisition. These results demonstrate a new role for MAGI proteins in the experience-dependent regulation of both subunit-specific AMPAR trafficking and behavioral plasticity *in vivo*.

## Materials and Methods

### Strains

Standard methods were used to culture *C. elegans*. The following strains were used: N2, *odIs1[P_glr-1_::SNB-1::GFP]*, *nuIs108[GLR-1(4KR)::GFP]*, *nuIs25[GLR-1::GFP]*, *odIs63[GFP::GLR-2]*, *osm-9(ky10), mec-4(u253)*, *magi-1(tm446)*, *magi-1(tm464)*, *glr-1(ky176)*, and *glr-2(ak10)*. The original *glr-1(ky176)* strain contains an out-of-frame deletion that removes most of the protein, including the C-terminal tail, all three transmembrane domains, the pore-lining domain, and the ligand binding domain [8]. This strain was reported to have a dominant Mec (Mechanosensation) phenotype. When we obtained this strain, we immediately backcrossed it and found that the Mec phenotype is due to a separate mutation that is closely linked to *glr-1*. After outcrossing, we reconfirmed the *ky176* deletion by PCR, and found that *glr-1(ky176)* mutants, while having the decreased reversals and nose-touch recessive phenotypes found in *glr-1(n2461)* mutants [9], moved well and were wild-type for the Mec phenotype.

Non-integrated transgenic strains were isolated after microinjecting MAGI-1::mRFP fusion constructs (80 ng/µl) or cosmid T22B3 (15 ng/µl) using *ttx-3::gfp* or *ttx-3::mrfp* (a gift from O. Hobert) as a marker. Several independently derived lines were examined for each fusion construct, and co-localization was quantified from two representative strains.

### Sequence Analysis

Percent identity between *C. elegans* MAGI-1 and selected mammalian MAGI proteins (human MAGI-1α,2 & 3, mouse and rat MAGI-2) was calculated using DNA Strider1.4f1. For protein alignment, the diagonals method was used with a 6-aa block length, a mismatch penalty of “smaller (1)”, a gap penalty of “medium (2)”, and the weighting was calculated according to BLOSUM62. The alignment was done using ClustalW (http://www.ebi.ac.uk/Tools/clustalw/).

### Mechanosensory Environment

To expose animals to mechanostimulation, we adapted a mass training protocol from Rose et al [26]. Multiple animals (20–30) were placed on 6 cm cultivation dishes inside a secondary plastic container at 25°C, and then dropped ∼4 cm once a minute for 15 minutes. This protocol was repeated at one-hour intervals 7 times during the first larval period. Animals were then left unstimulated for 24 hours, and observations were made on young adults. To generate an environment deprived of mechanical stimulation, animals were raised on plates in an isolated plastic container buffered from vibration by four rings of sorbothane (each ring is 3 cm high, 6 cm wide; AudioQuest).

### Quantification of puncta

Fluorescent images of fluorescent fusion proteins were observed using a Zeiss Axioplan II and a 100× (NA = 1.4) PlanApo objective. Imaging was done with an ORCA charge-coupled device (CCD) camera (Hamamatsu, Bridgewater, NJ) using IPLab software (Scanalytics, Inc, Fairfax, VA). Exposure times were chosen to fill the 12-bit dynamic range without saturation. Maximum intensity projections of z-series stacks were obtained, and out-of-focus light was removed with a constrained iterative deconvolution algorithm (IPLabs). Nematodes were visualized as previously described [20].

Cluster outlines and mean intensity were automatically calculated for fluorescent signals that were two standard deviations above the unlocalized baseline using ImagePro. Cluster number was calculated by counting the average number of clusters per 100 microns of dendrite length. Average mean puncta intensities were calculated for each animal, and then for the population of animals. Comparisons of integrated transgenic strains (*nuIs25*, *odIs1* and *nuIs108*) with and without *magi-1(tm446)* were done on sibling strains derived from the same heterozygous hermaphrodite. All strains were raised and stimulated (where appropriate) at 25°C in parallel. Each set of observations was performed at least twice on different sets of animals of the same genotype. Statistical tests were done assuming a two-tailed distribution of unequal variance, both on the quantification of puncta and data from the behavioral assays.

Colocalization between MAGI-1L::mRFP and GLR-1::GFP was performed by overlaying separate channels, and then classifying puncta as colocalized if greater than 50% of the pixels of an individual punctum in one channel overlapped with a punctum in the other channel. Non-overlapping puncta were also classified, with three classes (GLR-1 alone, MAGI-1 alone, and colocalized) being totaled for each animal. Classes were then normalized for the total number of puncta per animal. Data was pooled from three separate injection lines.

### Behavioral analyses

The reversal frequency of individual animals was assayed as previously described [27], but with some modifications. Three young adult hermaphrodites were placed on NGM plates in the absence of food. The animals were allowed to adjust to the plates for 6–7 minutes, and the number of spontaneous reversals for a single animal was counted over a 4-minute period. Again, all strains compared in a particular assay were raised and stimulated (where appropriate) at 25°C in parallel. Each assay was performed at least 3 times on different animals of the same genotype. The first two trials of spontaneous reversal frequency were done on animals of known genotype; however, those results were confirmed in trials blind to genotype, and all subsequent trials were done blind.

Habituation assays were performed in two ways. First, habituation to body touch was assayed using an eyebrow hair to stroke the animal transverse to the body length. The animals were stroked alternately on their anterior, between the pharynx and vulva, and on their posterior (tail). We assayed habituation by measuring the failure of an animal to reverse direction in response to touch. We counted the number of touches to the anterior body required before there was a failed response. We also counted the number of anterior touches required before there were two consecutive failures; this number was consistent with the first. In order to prevent contact between animals during the assay, three animals were put on each plate and allowed to recover from the move for at least 45 minutes. Only animals that stayed on the bacterial lawn throughout the assay were recorded.

Our second assay for habituation measured reversal magnitude. Single animals were put on each plate and allowed to recover for 2 hours. Each plate was then dropped from a height of 9 mm. Reversal magnitude was estimated as the number of quarter body bends executed in the response. Each plate was dropped 10 times. These experiments, as those above, were done blind to genotype in order to eliminate any bias in scoring. Both sets of habituation assays were performed over three separate trials on different animals of the same genotype.

### Coimmunoprecipitations

For testing MAGI-1/GluR interactions, MAGI-1S and MAGI-1L cDNA sequences were subcloned into pCMV expression vectors (Stratagene), which include a FLAG tag sequence. The GLR-1 (amino acids 876–972) and GLR-2 (amino acids 919–977) cytosolic tail sequences were subcloned into a CMV-based expression vector containing GST tag sequences (pDEST27, Invitrogen). COS-7 cells were cultured as described [28] and transiently cotransfected with FLAG and GST-tagged proteins using Lipofectamine Plus (Invitrogen). After 24 hours, transfected cells were lysed in 1 mL of cold modified RIPA (50 mM Tris-HCl pH 7.4, 125 mM NaCl, 0.25% NP-40) with protease inhibitors (Roche). Lysates were cleared of debris, and used for immunoprecipitations using anti-FLAG antibodies on beads (Amersham). Beads were washed in modified RIPA buffer, and bound proteins were eluted in SDS loading buffer, then analyzed by SDS-PAGE. Precipitated proteins were detected by Western blotting using polyclonal anti-FLAG or anti-GST primary antibodies and HRP-conjugated secondary antibodies. Exposed films were digitized and quantified using ImagePro. The amount of coimmunoprecipitated material was compared to “input” after adjusting for volumetric differences in loading. Similar results were observed in three independent transfection experiments.

### Yeast Two-hybrid

We performed yeast two hybrid experiments by placing GluR cytosolic tail sequences into the pDEST22 prey vector (Invitrogen). The resulting plasmid was cotransformed into yeast strain AH109 with a *C. elegans* cDNA library. Transformed yeast (∼5 million clones) were screened on –Leu –Trp –His dropout plates, followed by detection of β-gal staining. Three independent MAGI-1 cDNAs were identified.

### Anti-MAGI-1 staining

For examining endogenous MAGI-1, adults and embryos of the indicated genotype were fixed as described [29]. His-tagged fusion proteins of MAGI-1 WW domains (amino acids 260–323) were generated by fusing MAGI-1 WW domain sequences in frame to 6xHis of pET28b(+) (Invitrogen). The resulting plasmid was introduced into *E. coli* BL21, expression was induced, and fusion protein was recovered on nickel-containing resin after lysis. Purified protein was injected into rabbits to generate polyclonal antibodies (Pocono Rabbit Farm and Lab, Inc., PA). Fixed tissue was immunostained with anti-MAGI-1 antibodies at 1∶1000 titrations, and immunocomplexes were detected goat anti-rabbit secondary antibodies labeled with Cy3 (Jackson ImmunoResearch). Anti-DLG-1 staining was performed as described [30]. Images were captured as above.

### Transgenes

The MAGI-1S:: mRFP and MAGI-1L::mRFP transgenes were generated by introducing mRFP in frame after the relevant cDNA sequences lacking a stop codon. The resulting chimeric sequences were then placed downstream of the *glr-1* promoter in pV6 (a gift from V. Maricq).

### RNAi feeding experiments

Two kb of sequence containing the *magi-1* WW and PDZ domains (common to both isoforms) were subcloned into PD129.36 (a gift from A. Fire), and the resulting plasmid was introduced into HT115(DE3) *E. coli*. Feeding RNAi was then performed as described, using the N2 strain [31].

## Results

### Screens for regulators of AMPARs identified the MAGI-1 scaffolding molecule

The PDZ tail sequence of GLR-2 is important for the regulation of GLR-2 synaptic localization [14]. While the GLR-2 subunit does not form functional channels in the absence of GLR-1, most of the fast-activating current in the command interneurons requires GLR-2, suggesting that the predominant species of AMPARs are GLR-1/GLR-2 heteromers [10]. We reasoned that GLR-1/GLR-2 heteromers might be regulated by PDZ domain proteins that interact with the GLR-2 cytosolic tail, and thus took two approaches to identify such proteins. First, we conducted a yeast two-hybrid screen using GLR-2 cytosolic tail sequences as bait. Second, we screened a collection of mutants deleted for known PDZ-containing genes to identify proteins that affect GLR-1::GFP localization (we used the GLR-1::GFP transgene because the GLR-2::GFP transgene is not as well expressed). Both approaches converged on the synaptic scaffolding molecule MAGI-1.

**Figure 1:**
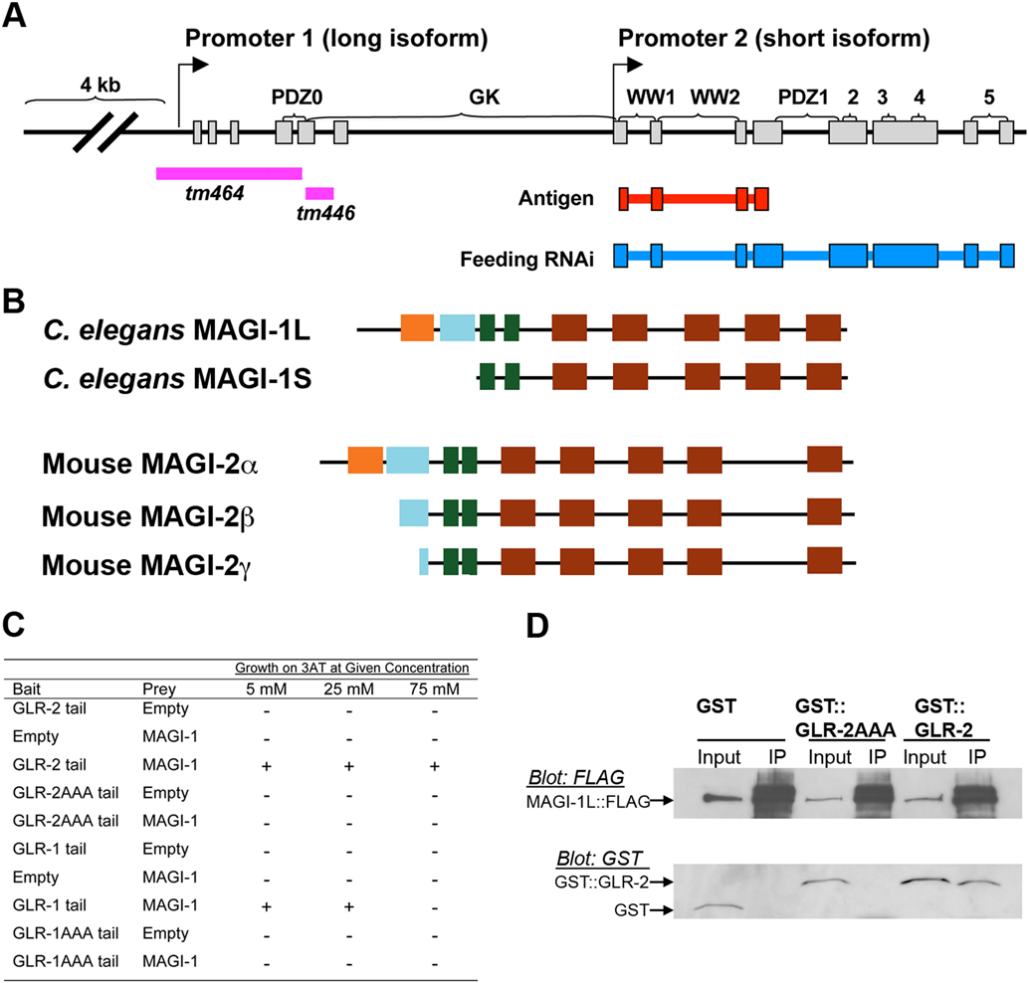
The *magi-1* gene encodes two MAGI/S-SCAM isoforms, the longer of which interacts with GLR-2. (A) *magi-1* is transcribed independently from exon 1 and exon 7 (arrows). Pink bars indicate genomic regions deleted in *tm446* and *tm464*. Based on RT-PCR results, both *magi-1(tm446)* and *magi-1(tm464)* animals transcribe a truncated long isoform: *tm446* deletes part of the guanylate kinase domain and inserts 5 extra amino acids, and *tm464* begins transcription at exon 4, resulting in a protein lacking PDZ0. Expression of the short isoform is not affected in either mutation. Graphic modified from Wormbase (www.worbase.org). (B) The domain topology of *C. elegans* MAGI-1 splice variants (S and L) are similar to splice variants of the mouse neuronal MAGI-2α, β and γ. The five canonical PDZ domains are shown in purple, the non-canonical PDZ0 is pink. The guanylate kinase domain is drawn in blue, and the two WW domains are green. (C) Table of yeast two-hybrid interactions for strains with the indicated bait and prey plasmids, plus the GAL4-driven reporters HIS3 and βGal. Growth on –His media containing 3-AT at the indicated concentration, plus βGal activity detected on a lift assay, are indicated by “+.” (D) COS-7 cells were transfected with FLAG-tagged MAGI-1L. Either GST, GST fused to the GLR-2 cytosolic domain (GST::GLR-2), or GST fused to the same domain with a mutated PDZ recognition motif (GST::GLR-2AAA) were cotransfected. Protein complexes were collected by immunoprecipitation using anti-FLAG antibodies. “Input” indicates 1.8% of the material used for immunoprecipitation. The “IP” lane indicates 25% of the material collected by immunoprecipitation. For each gel, the top half was immunoblotted using anti-FLAG antibodies, whereas the bottom half was immunoblotted using anti-GST antibodies. Greater than 90% of MAGI-1L was immunoprecipitated in each reaction; total amounts in each reaction varied less than 10%. Approximately 10% of GLR-2cd coimmunoprecipitated with MAGI-1L, whereas only 0–2% (variation from 3 separate blots) of GLR-2AAA coimmunoprecipitated. No detectable GST ever coimmunoprecipitated.

The *magi-1* locus encodes two independently transcribed long and short isoforms, which are similar in sequence and identical in domain topology to mammalian MAGI/S-SCAM proteins [4]. MAGI-1S (short isoform) contains two WW domains followed by five PDZ domains, whereas MAGI-1L (long isoform) contains the same sequences as MAGI-1S plus an additional PDZ domain and guanylate kinase (GuK) domain (Fig. 1A,B; Fig. S1). This arrangement is similar to mammalian MAGI-2 (Fig. 1B), which has three splice variants [3]–[5]. The mutants identified in our candidate screen delete sequences specific to the long isoform (Fig. 1A; Fig. S1); thus, we have focused our efforts on MAGI-1L.

### The GLR-2 AMPAR interacts with the MAGI-1L isoform

We also independently identified MAGI-1 in a yeast two-hybrid screen using the GLR-2 carboxy terminus as bait. When we coexpressed MAGI-1 prey and the GLR-2 carboxy-terminal domain (GLR-2 tail) bait in yeast that contain both *lacZ* and *HIS3* reporters, we detect growth on –His media and β-galactosidase activity, even in the presence of high concentrations of the HIS3 inhibitor 3-AT (Fig. 1C). We did not detect interaction in yeast strains that express either construct alone. To determine if the last three amino acids of GLR-2 (TLF), which match a PDZ recognition motif, are necessary for this interaction, we generated a bait, GLR-2AAA, in which these three amino acids were mutated to alanines. We could detect no interaction between MAGI-1 prey and GLR-2AAA bait. GLR-1 also has a PDZ binding motif at its C-terminus; therefore, we considered the possibility that MAGI-1L also interacts directly with GLR-1. We coexpressed MAGI-1 prey and the GLR-1 carboxy-terminal domain (GLR-1 tail) as bait, and found that MAGI-1 and the GLR-1 tail sequences interact, but weakly, as the interaction could be suppressed by 3-AT (Fig. 1C). This weak interaction also required the last three amino acids of GLR-1, as an analogous GLR-1AAA mutant did not interact.

To confirm and extend these results, we tagged MAGI-1L with FLAG, and the *glr-1* and *glr-2* cytoplasmic domains with GST, and co-expressed them in COS-7 cells. Using anti-FLAG agarose beads, we were able to co-immunoprecipitations MAGI-1L::FLAG and GST::GLR-2 from cell lysates (Fig. 1D). The interaction between MAGI-1L and the GLR-2 tail is strongly dependent on the PDZ binding motif in the GLR-2 cytoplasmic domain: mutating the motif to alanines (i.e., GST::GLR-2AAA) decreased the interaction by an order of magnitude (averaged over three independent trials). We also detected an interaction between MAGI-1L and the GLR-1 carboxy-terminus; however, this interaction was much weaker (data not shown). Interestingly, similar experiments with the MAGI-1 short isoform did not show an interaction with either GLR-1 or GLR-2 (data not shown), suggesting that the interaction might be specific to MAGI-1L. Unfortunately, the limited coexpression of GLR-1, GLR-2, and MAGI-1 in only six neurons *in vivo* (AVAL, AVAR, AVDL, AVDR, RMDVL, RMDVR) has precluded us from testing whether an AMPAR/MAGI-1L complex can be coimmunoprecipitated directly from *C. elegans*. Nevertheless, our two independent assays demonstrate that MAGI-1L can directly interact with the *C. elegans* AMPAR GLR-2 via its PDZ recognition motif.

### AMPARs are co-expressed with MAGI-1L in the reversal circuit

**Figure 2:**
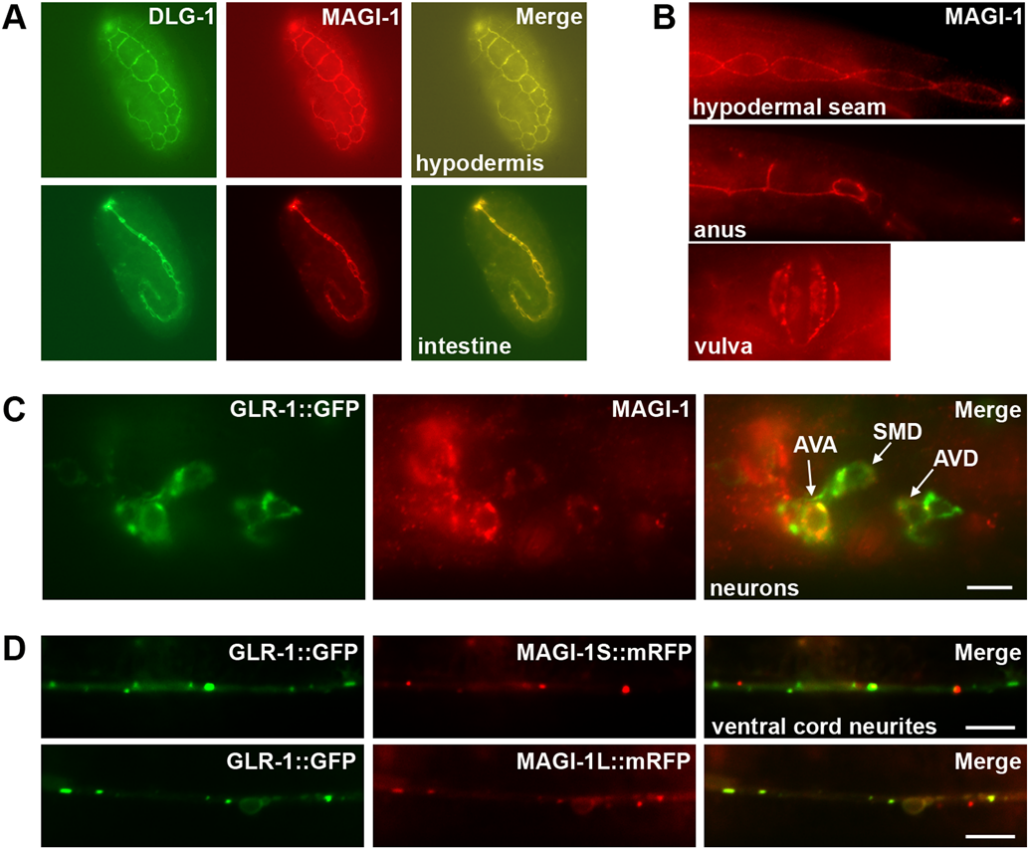
MAGI-1 is localized to adherens junctions and synapses. (A) As detected by fluorescence immunohistochemistry, MAGI-1 (red) co-localizes with DLG-1 (green) in a sub-set of hypodermal tissue (top) and intestine (bottom) during development. Shown in tadpole stage embryos. (B) MAGI-1 persists in a subset of epidermal tissues, including the hypodermal seam cells, the intestine (not shown), and the anus throughout development. An early larval stage is shown, anterior to the left, ventral side down. MAGI-1 is also localized to junctions in the developing and fully formed vulva (ventral aspect). (C) MAGI-1 and GLR-1::GFP were detected by fluorescence immunohistochemistry. Lateral view of the head showing MAGI-1 (red) labeling GLR-1::GFP-expressing cells (green). Anterior is left and dorsal is up. MAGI-1 was detected in the neurons AVA, AVD, and SMD (indicated by white arrows), as well as RMD and RMDV (not shown). (D) GFP and mRFP fluorescence detected in live animals. Ventral view of interneurons expressing GLR-1::GFP (green) and a C-terminal mRFP fusion (red) to either the short (MAGI-1S, top panels) or long (MAGI-1L, bottom panels) isoform of MAGI-1 (expressed from the *glr-1* promoter). Anterior is to the left. Bars, 5 µm.

If MAGI-1L and the *C. elegans* AMPARs interact *in vivo*, then they should be expressed in the same cells: the command interneurons of the reversal circuit. To examine MAGI-1 expression, we generated an antibody to the region containing the WW domains, found in both isoforms. This antibody labeled both neurons and epithelial junctions in immunofluorescence experiments. Labeling was severely reduced or absent in wild-type animals that were exposed to *magi-1* dsRNA, while labeling with an antibody to a similar MAGUK family scaffolding molecule, DLG-1 (a component of the adherens junctions), was unaffected in all of these same animals (Fig. S2A). By contrast, animals that were exposed to control dsRNA (the pPD129 vector) co-labeled with antibodies for both MAGI-1 and DLG-1 (data not shown). Within the hypodermis and intestine, there was a high degree of colocalization between MAGI-1 and DLG-1 (Fig. 2A). The anus and vulva also were labeled in larval and adult animals (Fig. 2B). Finally, MAGI-1 expression was observed in some neurons in the head, including a subset of the GLR-1::GFP-expressing command interneurons (Fig. 2C); these neurons (AVA and AVD) also express GLR-2 [11]. The command interneurons that govern locomotion reversal are directly connected to mechanosensory neurons and to each other via gap junctions and AMPAR-populated synapses (Fig. S3). We found that MAGI-1 is expressed in the backward but not the forward command interneurons; we did not detect MAGI-1 expression in any of the mechanosensory neurons (Fig. 2C). The expression of genomic *magi-1::gfp* and *magi-1::rfp* chimeric transgenes, which contain 4 kb of upstream sequence and either the complete *magi-1* ORF or only the genomic *magi-1* long isoform sequences, yielded similar results (Fig. S2B–E).

We next examined whether MAGI-1 and GLR-1 were colocalized at the subcellular level. Our anti-MAGI-1 antibody detected MAGI-1 localization to puncta along the ventral cord (data not shown); however, MAGI-1 is expressed in more neurons than just those that express GLR-1, making analysis of colocalization in the ventral cord difficult. To help restrict our analysis to the GLR-1-expressing neurons, we expressed both MAGI-1 isoforms as mRFP chimeric proteins under the *glr-1* promoter; both substantially colocalized with GLR-1 (Fig. 2D).

### MAGI-1L regulates GLR-1 localization in response to prior mechanical stimulation

As little is known about the function of MAGI proteins *in vivo*, we set out to test the hypothesis that MAGI-1L regulates the localization and function of AMPARs. Indeed, in addition to identifying MAGI-1 via its interaction with GLR-2 in a yeast two-hybrid screen, we also independently identified *magi-1* while visually screening for AMPAR trafficking defects (using the *glr-1::gfp* transgene) in a collection of mutants deleted for known PDZ-containing genes. We found that a deletion mutation, *magi-1(tm446)*, which removes sequences that are present only in the long isoform (Fig. 1A) and that encode conserved amino acids from the GuK domain (Fig. S1), had reduced numbers of localized GLR-1 puncta. Interestingly, we noticed that this phenotype varied according to the level of prior mechanosensory stimulation experienced by the mutants (e.g., incidental vibrations and jostling of their culture dish during incubation).

**Figure 3:**
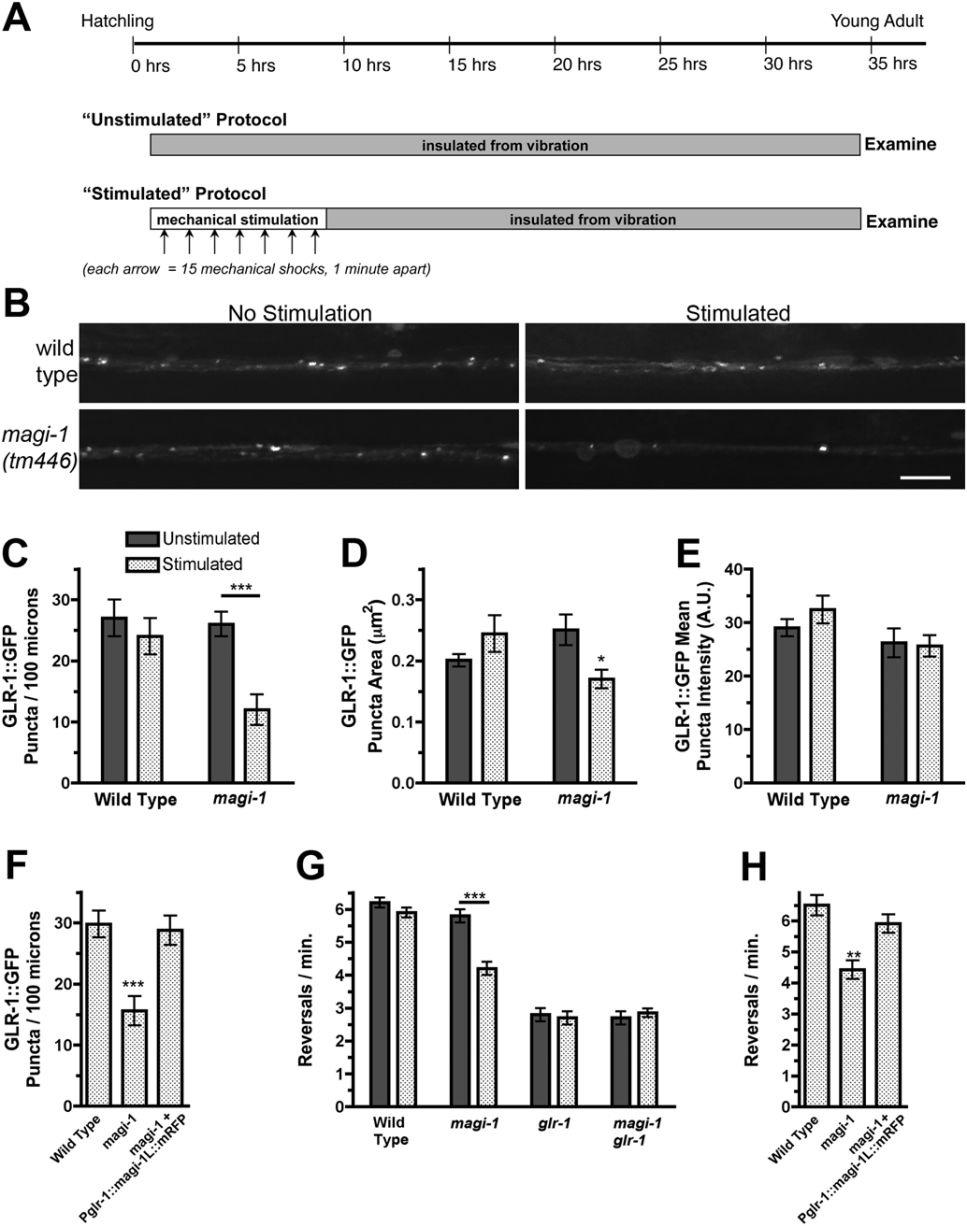
MAGI-1L regulates GLR-1 localization in response to prior experience. (A) Timeline for the stimulation protocol. Gray boxes indicate periods of vibration-free isolation, whereas the white box indicates a period of mechanostimulation. Each arrow indicates a set of 15 mechanical shocks delivered one minute apart. (B) GLR-1::GFP puncta in wild type and *magi-1(tm446)* mutants, both undisturbed animals (young adult) and animals mechanically stimulated the previous day. Ventral aspect, anterior is to the left. Bar, 5 µm. The mean (C,F) number per 100 microns, (D) size, and (E) intensity of GLR-1::GFP fluorescent puncta in the anterior cord was quantified (see Methods). (G,H) The mean spontaneous reversal frequency of animals is indicated. (C–H) Animals were either unstimulated (solid bars) or previously exposed to mechanical stimulation (stippled bars). Error bars indicate s.e.m. (C,D,G) ***p<0.0001, *p<0.01, Factorial ANOVA. (F,H) ***p<0.001, **p<0.01, One-way ANOVA followed by Dunnett's posthoc comparison to wild type, n = 15–25 for each genotype/condition. Data is pooled from three independent transgenic lines.

To examine the long-term consequences of prior mechanosensory stimulation on GLR-1 more fully, we adapted a previously described protocol [26]. We raised newly hatched animals in a regimented, mechanically stimulated environment by tapping their cultivation dishes once a minute for 15 minutes, repeating at one-hour intervals 7 times during the early larval period (Fig. 3A). As a control, we also raised animals in an environment deprived of mechanical stimulation, buffering cultivation dishes from vibration using sorbothane. After treatment, animals were isolated from vibration for 24 hours, and observations were made on young adult animals. Wild-type animals expressing an integrated GLR-1::GFP fusion showed only an slight, insignificant decrease in the number of GLR-1::GFP puncta in the interneurons if given prior stimulation (Fig. 3B,C). In contrast, we observed a large and significant reduction in the number of GLR-1::GFP puncta in *magi-1(tm446)* mutants that had been exposed to prior mechanical stimulation. Unstimulated *magi-1* mutants had slightly fewer GLR-1::GFP puncta than wild type, but the difference was not statistically significant. We also observed a reduction in the size of the remaining GLR-1::GFP puncta in stimulated *magi-1(tm446)* mutants, although their intensity was unaffected (Fig. 3D,E).

The stimulation-dependent reduction in GLR-1::GFP puncta was recessive, and observed in *magi-1(tm446)*/deficiency hemizygotes (data not shown). Furthermore, the homozygous mutant phenotype was rescued both by a cosmid carrying the *magi-1* genomic locus and by a 13 kb genomic fragment of *magi-1* fused to mRFP (data not shown). Finally, another deletion allele of *magi-1*, *magi-1(tm464)*, also specific to the long isoform, behaved identically to *tm446*. These data, together with the finding that the *tm446* phenotype is recessive, are consistent with a loss of function.

If MAGI-1L regulates AMPARs directly, then it should function in the same cells as the receptors. To test for cell autonomous rescue of the GLR-1::GFP localization defect, we used the *glr-1* promoter to express MAGI-1L::mRFP in the command interneurons. We found that *P_glr-1_::magi-1L::mRFP*, which was only expressed in the GLR-1-expressing neurons, fully rescued the GLR-1 localization defects of *magi-1(tm446)* mutants (Fig. 3F), indicating that the long isoform/mRFP chimera is functional in the same cells as GLR-1.

In order to confirm that the decrease in GLR-1::GFP was not an artifact of overexpression, we assayed the spontaneous reversal frequency of wild-type and *magi-1(tm446)* animals. GLR-1 mediates spontaneous reversals in locomotion during foraging, and *glr-1* mutants have a reduced frequency of spontaneous reversals [10], [21], [32]. We compared the rate of spontaneous reversals of wild type, *glr-1(ky176)* nulls, *magi-1(tm446)* mutants, and *glr-1(ky176) magi-1(tm446)* double mutants in mechanically stimulated and unstimulated animals (Fig. 3G). The reversal frequency of wild-type animals was not significantly changed by prior mechanical stimulation. Unstimulated mutants for *magi-1(tm446)* performed similarly to wild type. However, when mutants were stimulated 24 hours prior to testing, the reversal frequency dropped to a level midway between that in wild type and in animals lacking GLR-1 altogether. This behavioral mutant phenotype was rescued both by a cosmid carrying the *magi-1* genomic locus and by a 13 kb genomic fragment of *magi-1* fused to mRFP (Fig. S4A). Moreover, we found that *P_glr-1_::magi-1L::mRFP*, which is only expressed in the GLR-1-expressing neurons, fully rescued the behavioral defects of *magi-1(tm446)* mutants (Fig. 3H).

Finally, we used mutants insensitive to mechanical stimuli to demonstrate that the reduction in reversal frequency occurs in response to prior activation of the interneurons by mechanosensory neurons. Two separate sensory circuits detect touch in *C. elegans*. Body touch neurons express MEC-4, a mechanosensitive epithelial sodium channel subunit [33], [34]. Nose touch neurons express OSM-9, a TRP channel [35]. Mutations in *mec-4* and *osm-9* preserve sensory neuron morphology, but render animals unable to sense touch. Interestingly, there is a partial (25%) decrease in reversal frequency in *osm-9(ky10) mec-4(u253)* double mutants, suggesting that some of the spontaneous reversals are triggered by mechanosensory events as animals explore their environment. Nevertheless, a significant level of spontaneous reversals remains in these mutants when compared to animals that lack *glr-1* receptors (Fig. S4B). We compared *osm-9(ky10) mec-4(u253)* double mutants to *magi-1(tm446) osm-9(ky10) mec-4(u253)* triple mutants with and without prior mechanical stimulation, and found that the reversal frequency of both strains were identical under all conditions (Fig. S4B). Thus, removing touch sensation eliminates the effect of prior stimulation on the behavior of *magi-1* mutants, suggesting that the stimulation-dependent decrease in reversal frequency is mediated by mechanosensation.

### MAGI-1L does not regulate the total number of synapses

**Figure 4:**
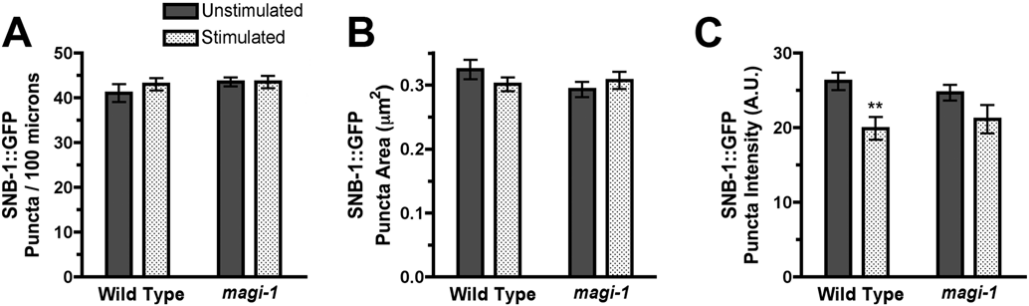
MAGI-1L does not regulate the total number of synapses. The mean (A) number per 100 microns, (B) size, and (C) intensity of SNB-1::GFP (synaptobrevin) fluorescent puncta in the anterior cord was quantified. Animals were either unstimulated (solid bars) or previously exposed to mechanical stimulation (stippled bars). Error bars indicate s.e.m. **p<0.001, Factorial ANOVA. N = 20–30 for each genotype/condition.

The decrease observed in the number of GLR-1 puncta could be due to a reduction in the total number of synapses. Alternatively, the number of synapses could remain constant, but fewer of them could contain GLR-1. To test these possibilities, we examined the presynaptic marker synaptobrevin using a synaptobrevin::GFP (SNB-1::GFP) fusion [20]. The ventral cord is so dense with synapses that labeling all of them with presynaptic SNB-1::GFP precludes any analysis of individual synapses. To get around this problem, we and others have visualized specific synapses by expressing SNB-1::GFP using cell-type specific promoters. A large fraction of the command interneuron synapses in the ventral cord are between different command interneurons themselves (Fig. S3); thus, a large number of the GLR-1-containing synapses can be observed by expressing the SNB-1::GFP reporter with the *glr-1* promoter [20]–[25]. We found that the changes in GLR-1 puncta observed in stimulated *tm446* mutants were not due to an overall decrease in the number or size of synapses, as the number of presynaptic boutons labeled with SNB-1::GFP did not change (Fig. 4A,B). We did observe a decrease in the intensity of the SNB-1::GFP-labeled synapses 24 hours after stimulation; however, it was present in both wild type and *tm446* mutants (Fig. 4C). These findings indicate that MAGI-1L regulates the abundance of GLR-1-containing AMPARs at mechanosensory synapses depending on the mechanosensory environment in which the animals were raised. MAGI-1L is acting in stimulated animals to prevent the removal of AMPARs from synapses.

### Ubiquitination is required for GLR-1 removal in response to stimulation

**Figure 5:**
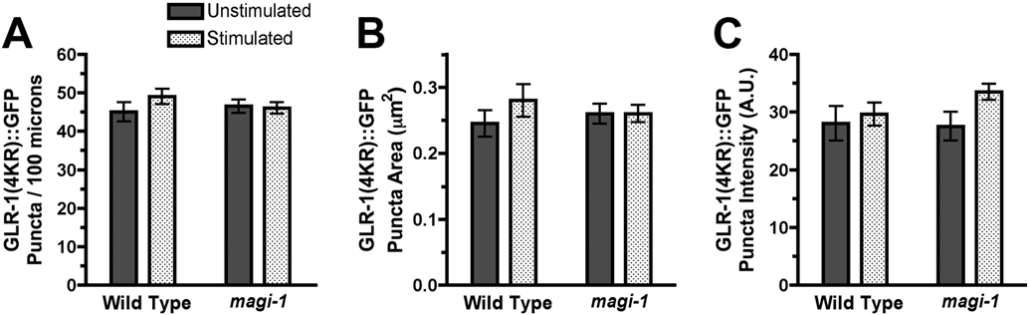
Ubiquitination is required for GLR-1 removal in response to stimulation. The mean (A) number per 100 microns, (B) size, and (C) intensity of GLR-1(4KR)::GFP (GLR-1 with mutated ubiquitination sites) fluorescent puncta in the anterior cord was quantified. Animals were either unstimulated (solid bars) or previously exposed to mechanical stimulation (stippled bars). Error bars indicate s.e.m. N = 20–25 for each genotype/condition.

How does prior stimulation result in GLR-1 removal? Ubiquitination is a common mechanism for regulating protein abundance by targeted proteolysis. It has previously been shown that a GLR-1::GFP construct in which four lysines in the C-terminal cytoplasmic domain have been mutated to arginine, GLR-1(4KR)::GFP, is correctly localized to synapses, but is unable to be ubiquitinated, and thus accumulates in the interneurons [25]. We used GLR-1(4KR)::GFP to assay whether ubiquitination is required for the decrease in GLR-1 levels in stimulated *magi-1* mutants. We found that the loss of GLR-1::GFP puncta in previously stimulated *magi-1(tm446)* mutants requires ubiquitination of the GLR-1 subunit, as the number of puncta in *magi-1(tm446)* mutants expressing GLR-1(4KR)::GFP does not decrease (Fig. 5A). Size and intensity also were unchanged (Fig. 5B,C). These results indicate that ubiquitination of the GLR-1 subunit is required in order for prior mechanical stimulation to result in the loss of GLR-1::GFP puncta in *magi-1(tm446)* mutants.

### MAGI-1L scaffolding molecules regulate AMPAR trafficking through GLR-2

**Figure 6:**
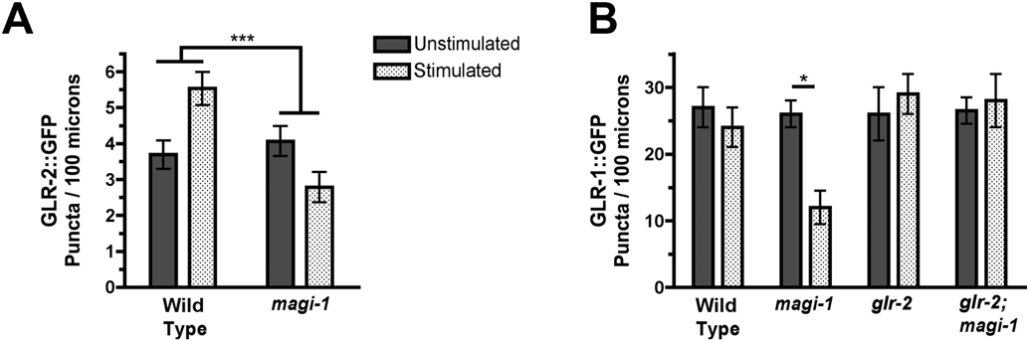
MAGI-1L regulates AMPAR trafficking through the GLR-2 subunit. (A) The mean number of GLR-2::GFP puncta per 100 microns in the anterior cord was quantified for animals of the indicated genotype either unstimulated (solid bars) or previously exposed to mechanical stimulation (stippled bars). (B) The mean number of GLR-1::GFP puncta is indicated. Error bars indicate s.e.m. *p<0.01, ***p<0.0001, Factorial ANOVA. N = 20–30 for each genotype/condition.

To determine whether MAGI-1 regulates GLR-2 *in vivo* as it does GLR-1, we used a transgene that expresses a full length GLR-2::GFP [10]. While not as well expressed as GLR-1::GFP, we found that GLR-2::GFP is nevertheless localized to puncta along the ventral cord in both stimulated and unstimulated wild-type animals, and stimulation leads to an increase in GLR-2 puncta number 24 hours later (Fig. 6A). When we expressed GLR-2::GFP in *magi-1(tm446)* mutants, we instead found a slight decrease in GLR-2::GFP puncta number in stimulated animals, similar to that observed for GLR-1::GFP puncta number in stimulated *magi-1(tm446)* mutants. Thus, GLR-2 localization, unlike GLR-1 localization, is increased in wild-type animals exposed to prior mechanical stimulation. Moreover, the effect of prior experience on GLR-2 localization is modulated by MAGI-1L.

As MAGI-1L protein can more strongly interact with GLR-2 than GLR-1, we reasoned that MAGI-1L might be primarily regulating GLR-1/GLR-2 heteromers (via its interaction with GLR-2) rather than GLR-1 homomers. Thus, we tested whether the GLR-2 subunit is required for MAGI-1L to regulate GLR-1::GFP localization. As described previously, mutants for *magi-1*, when stimulated, have a reduced number of GLR-1::GFP puncta (Fig. 6B). By contrast, double mutants for both *magi-1* and *glr-2* (as well as *glr-2* single mutants) do not show a reduction in GLR-1::GFP puncta (Fig. 6B), suggesting that GLR-2 is required for the effect of the *magi-1(tm446)* mutation on GLR-1 localization. Our results suggest that prior exposure to sensory stimulation can alter the subunit specific composition of AMPARs within a glutamatergic sensory circuit, resulting in more GLR-1/GLR-2 heteromers.

### MAGI-1L and GLR-2 are necessary for long-term memory in the touch circuit

**Figure 7:**
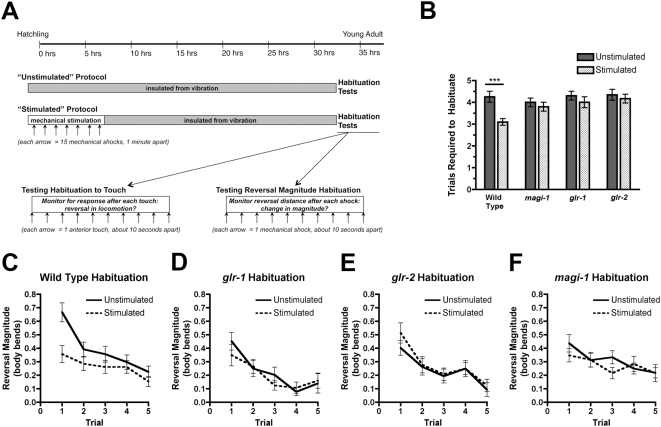
MAGI-1L and GLR-2 are necessary for long-term memory in the touch circuit. (A) Timeline for the stimulation protocol. (Top) Gray boxes indicate periods of vibration-free isolation, whereas the white box indicates a period of mechanostimulation. Each arrow indicates a set of 15 mechanical shocks delivered one minute apart. At the end of the protocol, animals are tested for habituation two ways: (lower Left) monitoring the number of touches (arrows) required before the animal begins to ignore the touch stimulus, and (lower right) monitoring the magnitude of the distance animals travel in reverse after individual, non-directional mechanical shocks (arrows) during a train of such shocks. (B) The mean number of trials required for animals (either previously stimulated or unstimulated) to habituate (i.e., fail to respond twice in a row to tap stimulation) during a train of mechanical stimulatory events is indicated. ***p<0.0001, Factorial ANOVA. (C–F) The mean magnitude of reversal (measured in body bends) in response to each mechanical shock, plotted out over a train of mechanical shocks, for (C) wild type, (D) *glr-1* mutants, (E) *glr-2* mutants, and (F) *magi-1* mutants. A solid line indicates unstimulated animals, whereas a dashed line indicates stimulated animals. Error bars denote s.e.m. N = 20–30 for each genotype/condition.

Finally, as a test of long-term memory, we observed whether prior stimulation alters the rate at which the reversal circuit habituates to subsequent stimulation [17]. Previous work has shown that prior mechanical stimulation, even after 24 hours, can depress the magnitude of the escape response to trains of subsequent mechanical stimuli; GLR-1 activity is required for this long-term memory [19]. We hypothesized that prior exposure to mechanical stimulation might also set the sensitivity of the interneurons to future mechanical stimulation or otherwise alter their ability to habituate to acute touch. As before, twenty-four hours after our stimulation protocol, we tested animals for their response to acute repeated mechanical stimulation. Then we determined the point at which an animal would fail to reverse in response to a touch on its anterior body during a train of touch trials (Fig. 7A, bottom left). Previously unstimulated animals took longer to habituate to body touch than animals exposed to prior stimulation (Fig. 7B).

In order to compare this method with previously published results, we also exposed animals to a train of mechanostimulatory events by repeatedly exposing their cultivation dish to a non-directional mechanical shock (dropping) and monitoring their reversal magnitude response (Fig. 7A, bottom right). We determined the reversal magnitude by measuring the number of quarter body bends executed by an animal during each single reversal in a train of trials in about a one-minute period. Previously unstimulated wild-type animals showed a strong habituation of reversal magnitude, with a rapid rate of decline in magnitude after several trials. By comparison, wild-type animals that had been stimulated the previous day showed a slow rate of decline in magnitude after several trials, similar to the published data (Fig. 7C, trials 1–5 are plotted).

We next used these two tests of habituation to assay null alleles of *glr-1* and *glr-2*, as well as *magi-1(tm446)* mutants. Mutants for *glr-1*, both stimulated and unstimulated, habituated to acute touch at the same rate as unstimulated wild-type animals (Fig. 7B). In addition, similar to previous reports [19], we also found that both stimulated and unstimulated *glr-1* mutants habituated their reversal magnitude in response to mechanical shock at the same rate (Fig. 7D). Mutants for *glr-2* and *magi-1L*, like mutants for *glr-1*, habituated to acute touch at the same slow rate regardless of prior stimulation (Fig. 7B), and did not display the stimulation-dependent effects on reversal magnitude that were observed in wild-type animals (Fig. 7E,F). Thus, mutations in *glr-1*, *glr-2*, and *magi-1L* appear to either prevent or erase the memory of prior mechanical stimulation. These results suggest that GLR-1, GLR-2, and MAGI-1L together modulate short-term habituation in response to long-term prior experience.

## Discussion

In this study, we identified a specific isoform of MAGI-1 as a novel regulator of experience-dependent plasticity and AMPAR trafficking. GLR-1 and GLR-2 AMPARs are expressed in the command interneurons of the *C. elegans* reversal circuit, where they mediate spontaneous reversals in locomotion during foraging. We adapted a protocol to expose animals to periodic, spaced mechanical stimulation, and then examined the long-term effects of this prior sensory experience on AMPAR function and subcellular localization. We found that the reversal frequency of wild-type animals is apparently kept constant regardless of prior mechanosensory experience. The number of GLR-1 puncta drops slightly, while there is a significant increase in the number of GLR-2 puncta. We found that the long isoform of MAGI-1 (MAGI-1L) is required to maintain the reversal frequency and modulate the number of GLR-1 and GLR-2 puncta in animals exposed to prior stimulation. Blocking the ubiquitin-dependent removal of GLR-1 can supersede the requirement for MAGI-1L during GLR-1 localization. Moreover, we found that MAGI-1L can interact with the cytosolic tail sequences of GLR-2, and that GLR-2 is required for the effect of the *magi-1(tm446)* mutation on spontaneous reversals and GLR-1 localization.

**Figure 8:**
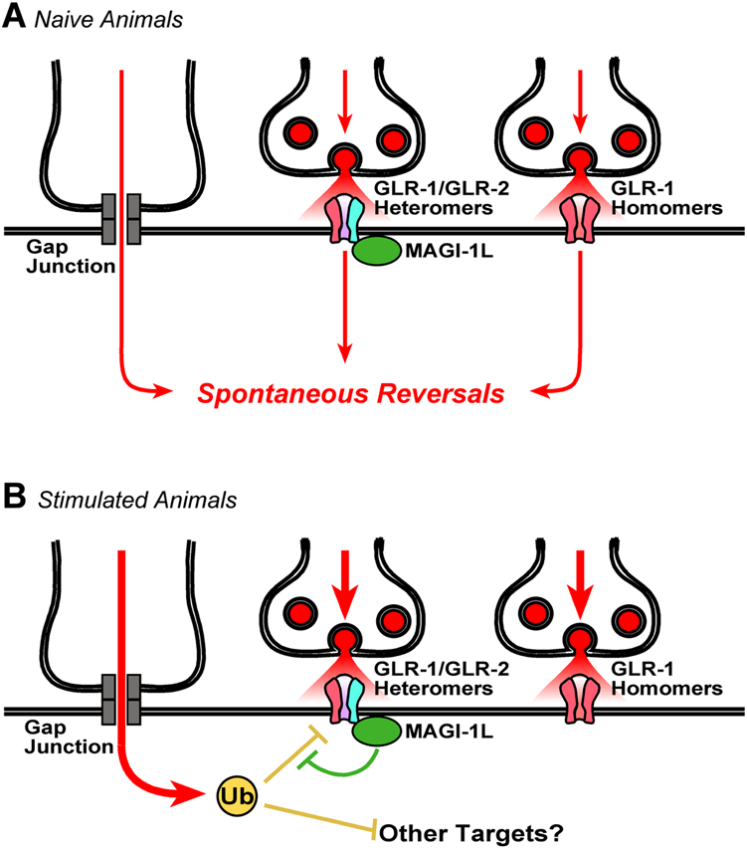
A model for the modulation of the reversal circuit by prior activity. (A) Reversal circuit synapses for wild-type animals likely contain heteromeric AMPAR channels comprised of GLR-1 (red) and GLR-2 (blue) subunits. MAGI-1L is shown as a green circle. The interneurons receive input (red arrows) from gap junctions and chemical synapses (synaptic vesicles are red circles). (B) Exposure to repeated mechanostimulation results in increased ubiquitination activity in the interneurons (only the activity from gap junctions is shown for simplicity). The removal of GLR-1/GLR-2 heteromers by ubiquitination (yellow T-bar) is prevented by MAGI-1L (green T-bar), which associates with the channel via its interaction with GLR-2. GLR-1 homomers appear to be immune to the effects of prior stimulation and MAGI-1L activity.

Based on our results, we propose the existence of a regulatory mechanism that maintains the constant, basal efficacy of the spontaneous reversal circuit regardless of prior sensory experience (Fig. 8). MAGI-1L is part of this regulatory mechanism, as mutations in this isoform result in a decrease in GLR-1 puncta and spontaneous reversal behavior for animals given prior stimulation. By contrast, naive, unstimulated animals do not require MAGI-1L function for reversal behavior or to maintain AMPAR puncta. MAGI-1L is only needed to modulate AMPAR localization and function in animals given prior stimulation. Thus, we can infer that prior stimulation must activate a mechanism that reduces AMPARs, and that MAGI-1L acts to counterbalance such a mechanism, thereby maintaining AMPAR localization in the face of variable mechanosensory experience.

What is the mechanism that MAGI-1L is counterbalancing? GLR-1 is removed by direct ubiquitination and endocytosis, and this process can be blocked if the ubiquitination sites on GLR-1 are mutated [25]. We found that by blocking GLR-1 ubiquitination, we could prevent the downregulation of GLR-1 puncta and the decrease in reversal behavior in stimulated *magi-1L* mutants, indicating that at least one of the mechanisms that MAGI-1L counterbalances is receptor ubiquitination. Interestingly, ubiquitination has been tied to multiple forms of synaptic plasticity, and increased neural activity results in a robust increase in ubiquitination in stimulated neurons [36]. Thus, proteins like MAGI-1L might protect certain factors like the AMPARs from degradation during periods of high neural activity.

Animals that are stimulated to reverse by nondirectional vibrations of their growth media (taps) presumably have increased levels of activity in the reversal circuit. We suggest that multiple, spaced bouts of such stimulation increase ubiquitination in the interneurons, and likely activate other signaling pathways as well (Fig. 8). The GLR-1-containing AMPARs are protected from such increased ubiquitination by MAGI-1L, which can directly bind to the GLR-1/GLR-2 heteromeric AMPARs via its association with the GLR-2 PDZ tail. Whether MAGI-1L protects GLR-1/GLR-2 AMPARs by preventing AMPAR ubiquitination, recruiting a deubiquitination enzyme to remove ubiquitin from the receptors, or preventing ubiquitinated AMPARs from being destroyed remains to be determined. In the absence of MAGI-1L, naive *magi-1* mutants have the normal complement of AMPAR puncta and reversals. By contrast, stimulated mutants remove many of the AMPAR puncta by ubiquitin-dependent pathways, resulting in decreased spontaneous reversals.

Are GLR-1/GLR-2 heteromers and GLR-1 homomers regulated differently? Surprisingly, wild-type animals exposed to prior mechanosensory activity show little change in the number of GLR-1 puncta, while simultaneously showing a significant increase in the number of GLR-2 puncta; this increase requires MAGI-1L. This finding suggests that MAGI-1L adds additional GLR-2 subunits to the ventral cord in response to prior mechanosensory experience. Interestingly, whereas MAGI-1L maintains a relatively high number of GLR-1 puncta, mutations that remove *glr-2* abolish this maintenance of GLR-1 localization, suggesting that GLR-1 homomers, unlike GLR-1/GLR-2 heteromers, are not apparently regulated by MAGI-1L. Thus, the presence of a GLR-2 subunit within the AMPAR channel appears to confer MAGI-1L-dependent regulation. These results are supported by the observed strong interaction of MAGI-1L with the GLR-2 C-terminal sequences, compared to the quite weak interaction of MAGI-1L with the GLR-1 C-terminal sequences. It should be noted that we cannot exclude the possibility that MAGI-1L regulates other accessory factors that assist GLR-1 and GLR-2 [37]. One prediction of our model is that MAGI-1L should preferentially associate with GLR-2-containing puncta in the ventral cord. Unfortunately, the GLR-2::GFP reporter is dim, and it is therefore likely that we are not visualizing all of the GLR-2-containing puncta. This drawback currently precludes a complete quantitative assessment of GLR-2/MAGI-1L colocalization.

Interestingly, MAGI-1 is only co-expressed with GLR-1 in the backward command interneurons AVA and AVD. It is thought that there is a competition between the forward and backward command interneurons to direct movement, and that the forward interneurons usually predominate, driving locomotion forward [12]. Presumably during a spontaneous reversal, the balance of activity in the circuit is briefly tipped towards the backward interneurons. This balance can be shifted in response to prior mechanostimulation [16]. While MAGI-1 likely only maintains AMPARs populating connections in the AVA and AVD backward interneurons, we suggest that this maintenance would have a significant enough impact on the forward-backward balance of activity in the circuit to alter the spontaneous reversal rate. Previous work indicates that the rate of spontaneous reversals is regulated by food and olfactory cues, and this regulation is critical for successful foraging and chemoattraction [38]–[40]. Thus, proteins like MAGI-1L probably help foraging animals maintain their spontaneous reversal behavior even in the face of periods of prior stimulation, allowing them to consistently navigate over long distances.

In prior studies, Rose et al. noted a decrease in GLR-1 puncta size in stimulated wild-type animals in a region of the posterior ventral cord (posterior to the vulva but anterior to the preanal ganglia) [19]. The posterior half of the animal contains only about 25% of the GLR-1 puncta, many of which belong to the PVC forward command interneurons [20], [41], [42]. We did not observe changes in GLR-1 puncta in stimulated wild-type animals; however, our studies focused on GLR-1 puncta in the anterior ventral cord, most of which are derived from backward command interneurons. It is likely that GLR-1 is regulated by neural activity in a different fashion in the forward versus the backward command interneurons, depending on the change in directional behavior required. We feel this is the likely explanation for the differences observed between our studies and those of Rose et al. Indeed, the restricted expression of MAGI-1 only to the backward command interneurons supports this view.

Whereas prior mechanostimulation does not affect spontaneous reversal frequency, prior stimulation can modulate the short-term habituation of the circuit to later stimuli [17], [18]. Naive animals become habituated during a single train of stimulations, as exhibited by a depression in reversal magnitude; this habituation can quickly recover. Multiple, spaced trains of prior stimulation, like our stimulation protocol, result in a long-lasting depression in reversal magnitude. Nevertheless, stimulated animals can still be further habituated; indeed, they habituate more rapidly than naive animals. We find that MAGI-1L, like GLR-1 and GLR-2, is also required for this form of long-term memory. Are the command interneurons the site of habituation for the circuit? While we cannot rule out that the command interneurons undergo habituation, there is evidence that the mechanosensory cells themselves can undergo habituation [16], [43]. For GLR-1, GLR-2, and MAGI-1L to be required in the command interneurons to modulate habituation in the mechanosensory neurons, one must posit that there is either a retrograde signal that mediates the modulation or that the command interneurons regulate a separate set of neurons, which in turn act as modulators of the mechanosensory neurons. Future studies will focus on the downstream signaling pathways that mediate this modulation.

Future studies should also reveal the role of the short isoform of MAGI-1, as different isoforms of scaffolding molecules can have disparate, sometimes contrary, functions [44]–[46]. Regardless, our current study indicates two important roles for the long isoform. On one hand, MAGI-1L is regulating AMPAR localization in response to prior experience, thereby maintaining a consistent spontaneous reversal frequency. On the other hand, MAGI-1L is also modulating short-term habituation in response to prior experience, thereby allowing animals to rapidly adjust to stimuli that they have previously encountered. This combination of functions allows the circuit to remain flexible in its response to acute mechanostimulation, while simultaneously maintaining consistency during long-term foraging and navigation towards chemoattractants.

## Supporting Information

Figure S1 C. elegans MAGI-1 is similar to vertebrate MAGI-1. Amino acid alignment of C. elegans (C.e.), Macaca mulata (M.m.), and human (H.s.) MAGI-1. Black highlighting indicates identities and gray highlighting indicates similarities in all three sequences. Overlines indicate specific protein domains (blue, PDZ; red, WW; gray, guanylate kinase). The dotted overlines indicate sequences that are missing in the mature mRNA produced from the indicated allele. The tm464 deletion removes the start of transcription and the first three exons. Nevertheless, a long isoform transcript was detected by RT-PCR, with the start of transcription at exon 5. Most PDZ0 sequences are not present in the final product. The tm446 deletion removes part of exon 4, including its 5 prime splice site. Splicing at a cryptic 5 prime splice site was detected RT-PCR. The product deletes a portion of the guanylate kinase domain, and inserts the amino acid sequence YGGTN.
(1.40 MB TIF)

Figure S2 MAGI-1 Expression and Subcellular Localization. (A) Double label fluorescence immunohistochemistry for DLG-1 (green) and MAGI-1 (red) on embryos that have been treated with a magi-1 feeding RNAi construct. Anti-MAGI-1 antibodies do not detect MAGI-1 protein in animals treated with the magi-1 RNAi construct, supporting the specificity of the antibody (compare to untreated wild-type embryos in Figure 1A). Note that the fluorescence observed in the pharyngeal lumen is due to nonspecific staining by the secondary antibody. As a control for tissue permeability, anti-DLG-1 antibodies detect junctional localization in the hypodermal tissues (top) and intestine (bottom) of magi-1(RNAi) embryos. Shown in three-fold stage embryo. (B) The expression reporter transgenes magi-1L::gfp and magi-1::rfp. Diagram labeling is as described for Figure 1. (C) The magi-1L:::gfp reporter transgene reflects the expression of the long isoform. Expression of MAGI-1L::GFP was detected in the pharynx, head neurons, ventral cord, and vulva of animals mosaic for the extrachromosomal array. (D) The magi-1::rfp reporter transgene reflects the expression of both isoforms. Neurons in the head are shown in animals that coexpress GLR-1::GFP. Expression of the reporter in the pharynx is out of the plane of focus. (E) Expression of MAGI-1L::RFP was also detected in (from left to right) the pharynx, intestine, vulval epithelia (L4 stage), and vulval muscle (adult stage) of animals mosaic for the extrachromosomal array. For pictures of the pharynx, it should be noted that neurons expressing MAGI-1 are out of the plane of focus. Unlike the MAGI-1L:::GFP reporter, which contains only the PDZ0 domain and is therefore unlocalized inside cells, the full length MAGI-1::RFP construct is localized to cellular junctions and punctate structures within cells.
(1.21 MB TIF)

Figure S3 A diagram of the mechanosensory circuit. Synaptic connections are black lines, with synapses as filled circles. Gap junctions are red lines, with junctions indicated by bars. Mechanosensory neurons are shown as gray circles. Touch-receptor components described in this manuscript (OSM-9 and MEC-4) are indicated. The command interneurons are shown as squares. Red neurons (AVA and AVD) coexpress GLR-1, GLR-2, and MAGI-1. The green neuron (PVC) coexpresses GLR-1 and GLR-2, but not MAGI-1. The blue neuron (AVB) only expresses GLR-1.
(1.44 MB TIF)

Figure S4 Additional behavioral data for magi-1(tm446) mutants. (A,B) The mean spontaneous reversal frequency of animals either unstimulated (solid bars) or previously exposed to mechanical stimulation (stippled bars) is indicated. Error bars denote s.e.m. (A) **p<0.01, One-way ANOVA followed by Dunnetts posthoc comparison to wild type, n = 15–25. Data for both the genomic cosmid rescue and the magi-1::rfp transgene rescue are pooled from three independent transgenic lines for each. (B) ***p<0.0001, Factorial ANOVA.
(0.19 MB TIF)
