# Supplementary figures and images for: MAGI-1 Modulates AMPA Receptor Synaptic Localization and Behavioral Plasticity in Response to Prior Experience
Source: PLoS One. 2009 Feb 26;4(2):e4613. doi: 10.1371/journal.pone.0004613 (PMC2645691; doi:10.1371/journal.pone.0004613)

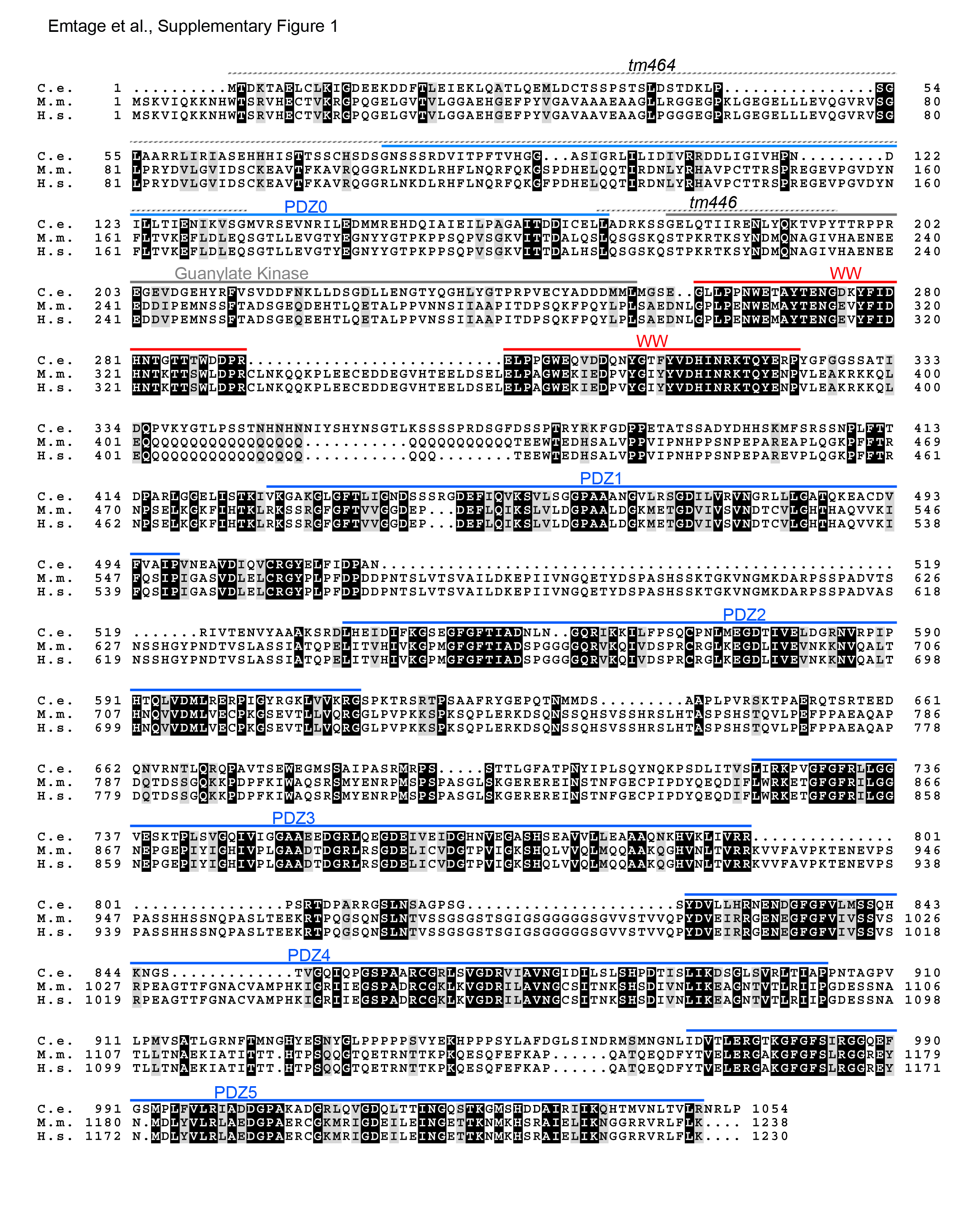

Supplement: Figure S1 — C. elegans MAGI-1 is similar to vertebrate MAGI-1. Amino acid alignment of C. elegans (C.e.), Macaca mulata (M.m.), and human (H.s.) MAGI-1. Black highlighting indicates identities and gray highlighting indicates similarities in all three sequences. Overlines indicate specific protein domains (blue, PDZ; red, WW; gray, guanylate kinase). The dotted overlines indicate sequences that are missing in the mature mRNA produced from the indicated allele. The tm464 deletion removes the start of transcription and the first three exons. Nevertheless, a long isoform transcript was detected by RT-PCR, with the start of transcription at exon 5. Most PDZ0 sequences are not present in the final product. The tm446 deletion removes part of exon 4, including its 5 prime splice site. Splicing at a cryptic 5 prime splice site was detected RT-PCR. The product deletes a portion of the guanylate kinase domain, and inserts the amino acid sequence YGGTN. (1.40 MB TIF) [file pone.0004613.s001.tif]

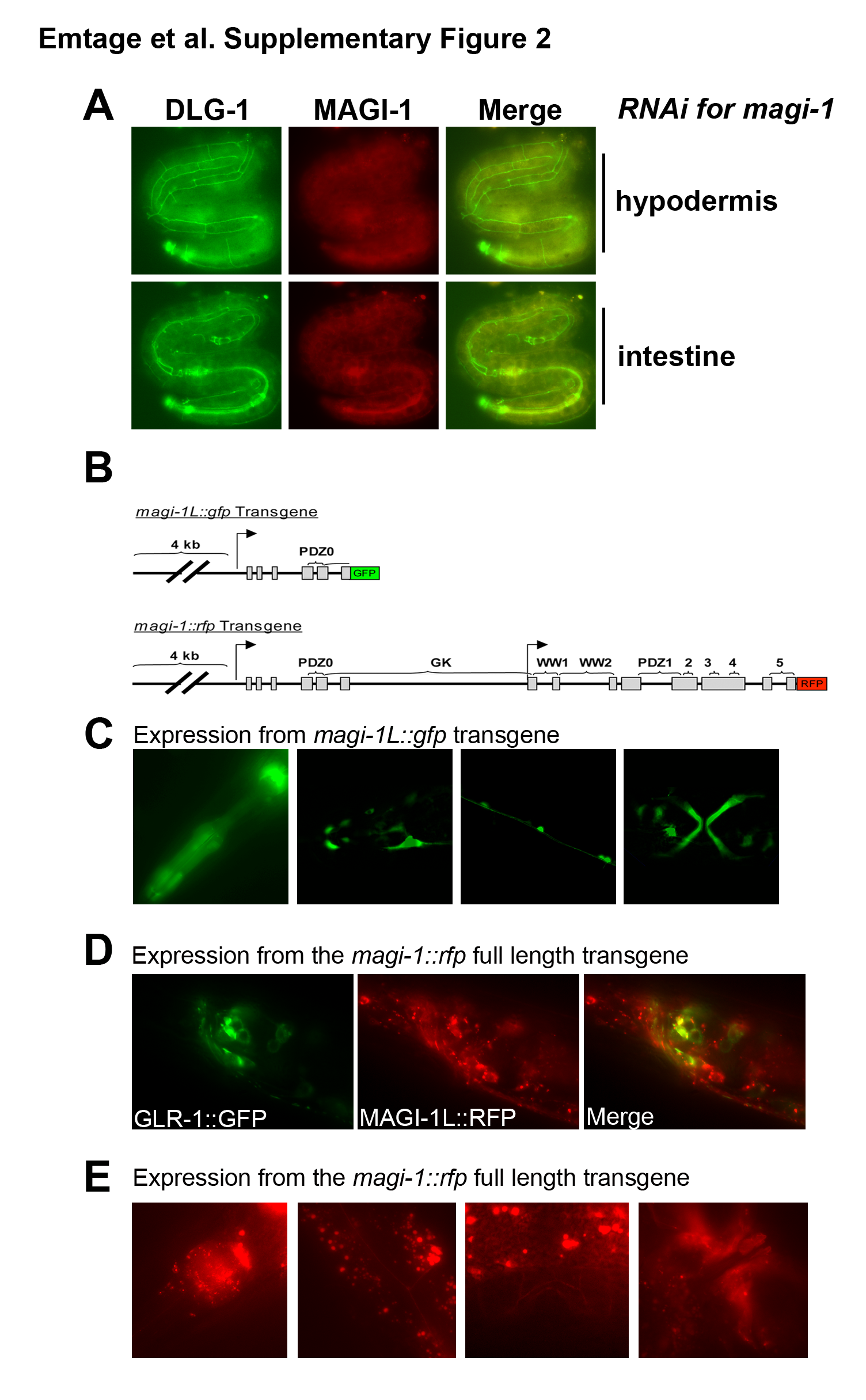

Supplement: Figure S2 — MAGI-1 Expression and Subcellular Localization. (A) Double label fluorescence immunohistochemistry for DLG-1 (green) and MAGI-1 (red) on embryos that have been treated with a magi-1 feeding RNAi construct. Anti-MAGI-1 antibodies do not detect MAGI-1 protein in animals treated with the magi-1 RNAi construct, supporting the specificity of the antibody (compare to untreated wild-type embryos in Figure 1A). Note that the fluorescence observed in the pharyngeal lumen is due to nonspecific staining by the secondary antibody. As a control for tissue permeability, anti-DLG-1 antibodies detect junctional localization in the hypodermal tissues (top) and intestine (bottom) of magi-1(RNAi) embryos. Shown in three-fold stage embryo. (B) The expression reporter transgenes magi-1L::gfp and magi-1::rfp. Diagram labeling is as described for Figure 1. (C) The magi-1L:::gfp reporter transgene reflects the expression of the long isoform. Expression of MAGI-1L::GFP was detected in the pharynx, head neurons, ventral cord, and vulva of animals mosaic for the extrachromosomal array. (D) The magi-1::rfp reporter transgene reflects the expression of both isoforms. Neurons in the head are shown in animals that coexpress GLR-1::GFP. Expression of the reporter in the pharynx is out of the plane of focus. (E) Expression of MAGI-1L::RFP was also detected in (from left to right) the pharynx, intestine, vulval epithelia (L4 stage), and vulval muscle (adult stage) of animals mosaic for the extrachromosomal array. For pictures of the pharynx, it should be noted that neurons expressing MAGI-1 are out of the plane of focus. Unlike the MAGI-1L:::GFP reporter, which contains only the PDZ0 domain and is therefore unlocalized inside cells, the full length MAGI-1::RFP construct is localized to cellular junctions and punctate structures within cells. (1.21 MB TIF) [file pone.0004613.s002.tif]

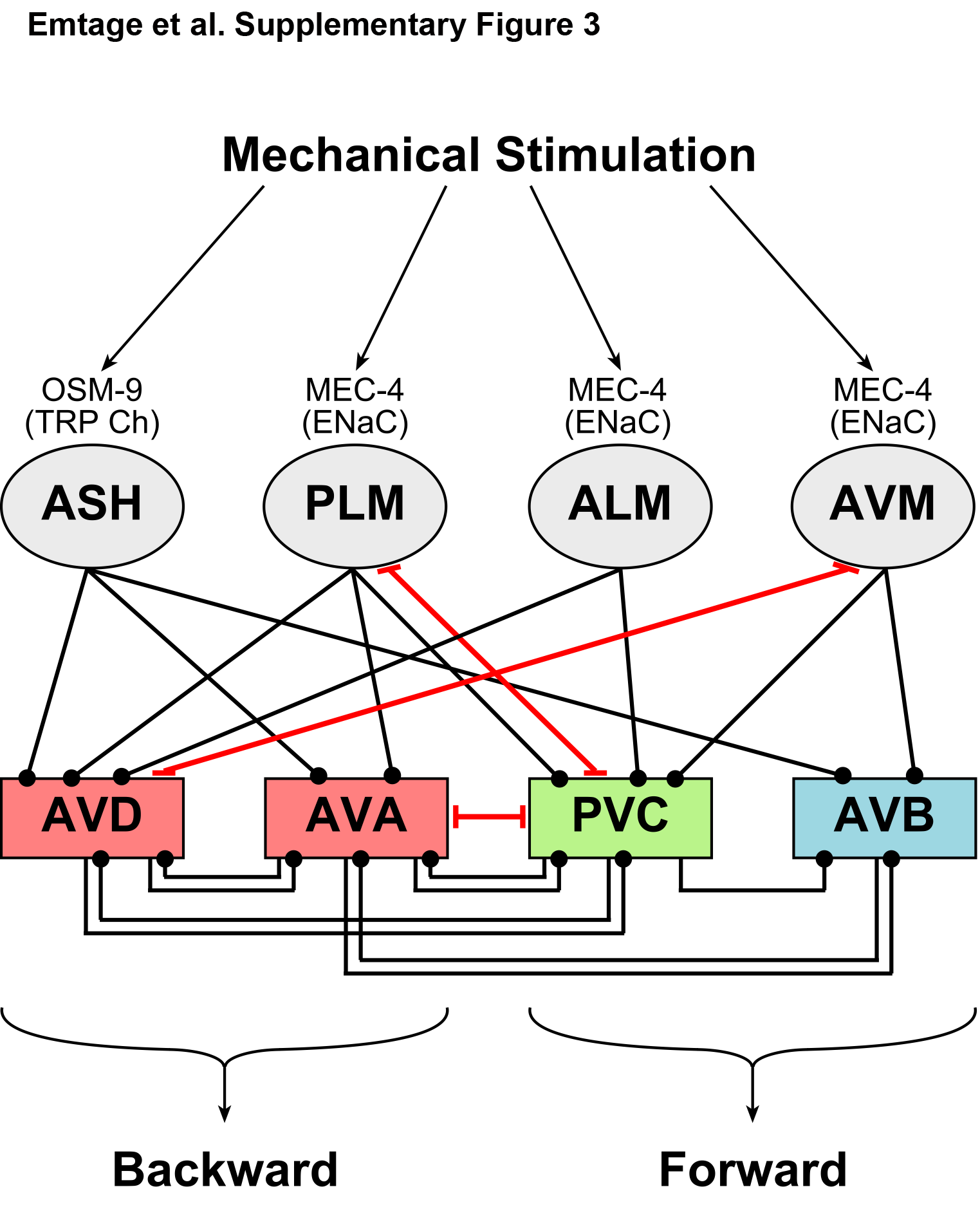

Supplement: Figure S3 — A diagram of the mechanosensory circuit. Synaptic connections are black lines, with synapses as filled circles. Gap junctions are red lines, with junctions indicated by bars. Mechanosensory neurons are shown as gray circles. Touch-receptor components described in this manuscript (OSM-9 and MEC-4) are indicated. The command interneurons are shown as squares. Red neurons (AVA and AVD) coexpress GLR-1, GLR-2, and MAGI-1. The green neuron (PVC) coexpresses GLR-1 and GLR-2, but not MAGI-1. The blue neuron (AVB) only expresses GLR-1. (1.44 MB TIF) [file pone.0004613.s003.tif]

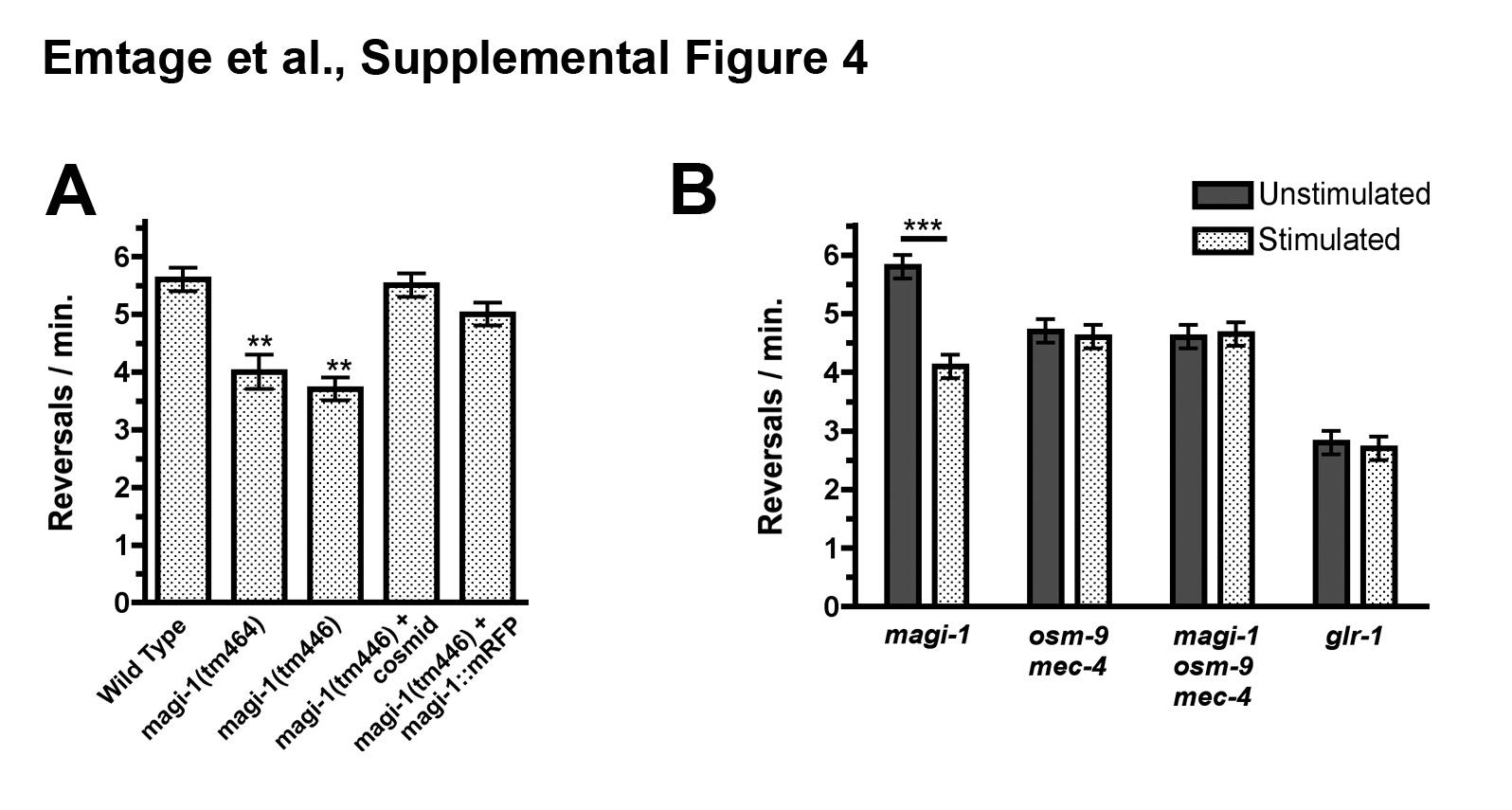

Supplement: Figure S4 — Additional behavioral data for magi-1(tm446) mutants. (A,B) The mean spontaneous reversal frequency of animals either unstimulated (solid bars) or previously exposed to mechanical stimulation (stippled bars) is indicated. Error bars denote s.e.m. (A) **p<0.01, One-way ANOVA followed by Dunnetts posthoc comparison to wild type, n = 15–25. Data for both the genomic cosmid rescue and the magi-1::rfp transgene rescue are pooled from three independent transgenic lines for each. (B) ***p<0.0001, Factorial ANOVA. (0.19 MB TIF) [file pone.0004613.s004.tif]
